# Real‐world drug use in asthma, chronic obstructive pulmonary disease, rhinitis, cough, and cold in Finland from 1990 to 2021: Association with reduced disease burden

**DOI:** 10.1002/clt2.12340

**Published:** 2024-04-01

**Authors:** Tiina Mattila, Vesa Jormanainen, Marina Erhola, Tuula Vasankari, Sanna Toppila‐Salmi, Fredrik Herse, Riikka‐Leena Leskelä, Tari Haahtela

**Affiliations:** ^1^ Department of Pulmonary Diseases Heart and Lung Center Helsinki University Hospital and Helsinki University Helsinki Finland; ^2^ Finnish Institute for Health and Welfare Helsinki Finland; ^3^ Ministry of Social Affairs and Health Helsinki Finland; ^4^ The Wellbeing Services County of Pirkanmaa Tampere Finland; ^5^ University of Turku Turku Finland; ^6^ Finnish Lung Health Association (FILHA) Helsinki Finland; ^7^ Department of Allergology Skin and Allergy Hospital Helsinki University Hospital and University of Helsinki Helsinki Finland; ^8^ Department of Otorhinolaryngology Kuopio University Hospital and School of Medicine Institute of Clinical Medicine University of Eastern Finland Kuopio Finland; ^9^ Nordic Healthcare Group Helsinki Finland

**Keywords:** asthma, COPD, health economics, public health, respiratory medications

## 1 INTRODUCTION

Asthma, chronic obstructive pulmonary disease (COPD), and allergic rhinitis are major long‐term airway diseases.^1^, ^2^, ^3^, ^4^, ^5^ Asthma appears in all age groups and COPD causes major morbidity and mortality particularly in smokers.^1^, ^2^, ^5^ Allergic rhinitis and other allergic conditions are often associated with asthma.^3^, ^4^, ^6^

In Finland, three nationwide respiratory programmes have been implemented since 1994: the Asthma Programme (1994–2004), the COPD Programme (1998–2007), and the Allergy Programme (2008–2018).^4^, ^6^ After the 1990s, the burden of asthma, COPD, and allergic conditions has decreased and the prevalence has stabilised.^4^, ^6^ Smoking has also decreased.^7^

International guidelines are available for asthma (since 1995), COPD (since 1997), and chronic rhinitis (since 2005).^1^, ^2^, ^3^

Inhaled short‐ and long‐acting β2 adrenoceptor agonists (LABA), muscarinic receptor antagonists (LAMA), and corticosteroids (ICS) have been used for first‐line treatment in asthma and COPD since the 1990s.^1^, ^2^, ^4^, ^8^ Intranasal corticosteroids have been used in rhinitis for decades.^3^

In the present study, we present consumption (sales) data of medication for asthma, COPD, rhinitis, cough, and cold from 1990 to 2021, and analyse the overall costs of asthma and severe COPD from 1996 to 2018.

Since 1988, the *Social Insurance Institution of Finland* (SII) and the *Finnish Medicines Agency* (Fimea) have jointly published the *Finnish Statistics on Medicine* (FSM), which includes all medications purchased in Finland.^8^

For sales statistics, medications are listed according to the *Anatomical Therapeutic Chemical* groups (R, Respiratory system).^9^ We report medication consumption for asthma and COPD (R03; inhaled (R03A‐B), systemic (R03DC), and molecular targeted medications (R03DX05, R03DX08–10)), nasal preparations (R01), systemic antihistamines (R06), and medications for cough and cold (R05; such as expectorants (R05C) and cough suppressants (R05D)).^9^

Medication consumption was followed using the unit *Defined Daily Doses* (DDD/1000 population/day) in the Finnish nationwide registries (FSM, SII). The results are presented as annual time series.

Cost analysis was made for asthma and severe COPD comparably to our previous work (data 1996–2018).^4^ This included only those individuals entitled to special reimbursed medication (same criterions 1996–2018).^4^

The population of Finland was accessed from *Statistics Finland,* and it increased from 5.0 million in 1990 to 5.5 million in 2021 (+10%).

**FIGURE 1:**
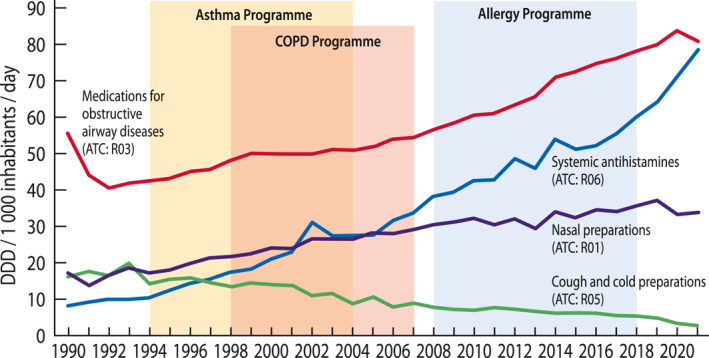
Consumption of medications for asthma, COPD, and allergic conditions and for cough and cold according to *Anatomical Therapeutic Chemical* statistics from 1990 to 2021 in Finland. The national Asthma Programme was run 1994–2004, COPD Programme 1998–2007, and Allergy Programme 2008–2018. Source: Finnish Statistics on Medicines 2021. COPD, chronic obstructive pulmonary disease.

Respiratory medications consumption increased from 98 in 1996 to 200 DDD/1000/day (+104%) in 2021. In 2021, there were altogether 590,000 patients (110/1000 inhabitants) who purchased medication for asthma or COPD. Systemic antihistamines were purchased by 480,000 persons (87/1000) and nasal preparations by 440,000 persons (79/1000) (Figure 1).

In 1990, there were 110,000 persons (21/1000) entitled to special reimbursed medication for persistent asthma or severe COPD. The respective number in 2021 was 290,000 (52/1 000, +164%). During the same period, mortality in COPD increased from 850 (0.15/1000) to 1100 (0.20/1000) but decreased in asthma from 98 to 61 deaths.

**FIGURE 2:**
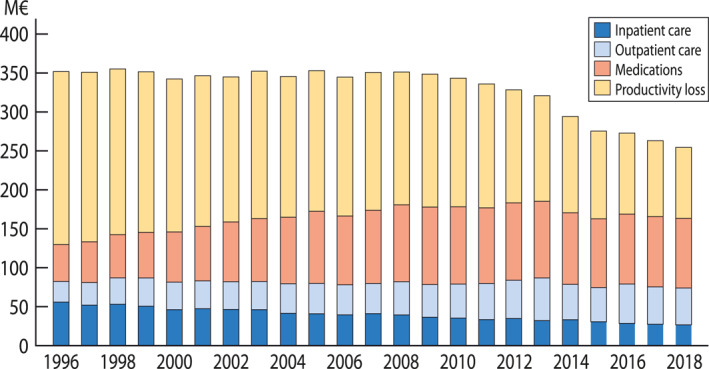
Asthma and COPD costs in Finland from 1996 to 2018. Direct healthcare costs consist of inpatient and outpatient care and medication (for medication, only those entitled for special reimbursement because of persistent asthma or severe COPD). Indirect costs consist of lost productivity caused by work absenteeism and disability pensions. COPD, chronic obstructive pulmonary disease.

The cost of specially reimbursed medication for asthma and severe COPD increased from 1996 up to 2005 but has been stable since (89 M€ in 2018). A major reduction was observed in the cost of inpatient care (from 55 to 26 M€; −47%) and in productivity loss (220 to 91 M€; −59%) (Figure 2).

Changes in population characteristics may have influenced the use of respiratory medication. In the adult population, body weight has steadily increased from 1980 to 2017. Smoking increased until 2017 when it started to decrease.^7^ The population is ageing, as the number of those aged ≥65 years almost doubled in 1990–2021, from 670,000 to 1,300,000.

In asthma, ICS as a first‐line medication made a breakthrough in the early 1990s, and the combination of ICS and LABA in late 1990s.^1^, ^4^ Adding LAMA in the combination was the next step to treat more severe asthma and COPD in the 2000s.^1^, ^2^, ^8^

Biological medication for asthma has been available in Finland since 2002 (first omalizumab). Their use has been marginal until the last few years and their costs are not included in the present calculations. Biologicals are expensive compared with inhaled medication, but their targeted use in severe asthma is indicated.^10^ The evidence for their cost‐effectiveness in Finland should be separately evaluated.

COPD was acknowledged as a major public health concern the late 1990s.^2^ Since the early 2000s, the focus in medication has been LABA and LAMA. Triple inhaled therapy (ICS + LABA + LAMA) has been increasingly used from the 2010s.^2^, ^8^

Most patents for inhaled medications for obstructive airway diseases expired in the 2000s.^8^ This probably explains in part why medication costs in asthma and COPD have not increased in Finland, although both consumed medications and the number of persons with asthma and COPD entitled to reimbursed medication have increased.^4^ Nevertheless, the overall burden and costs caused by obstructive airway diseases have decreased (Figure 2).

Most savings are best explained by improvements in diagnostics and early anti‐inflammatory (ICS) treatment of asthma along with the national Asthma Programme (1994–2004).^4^, ^6^

There was an increasing trend during decades in the prevalence of allergic rhinitis and other allergic conditions. This trend stabilised during the Allergy Programme (2008–2018).^6^, ^7^ Biological medication for asthma has been available in Finland since 2002 (first omalizumab). Their use has been marginal until the last few years and their costs are not included in the present calculations. Biologicals are expensive compared with inhaled medication, but their targeted use in severe asthma is indicated.^10^ The evidence for their cost‐effectiveness in Finland should be separately evaluated.^3^ Antihistamines became over‐the‐counter medications in the early 2000s,^8^ which have markedly increased their use.

Common cold is the most common cause for cough (13). If the underlying cause of cough is asthma or COPD, this should be targeted immediately.^1^, ^2^ The market for antitussive medications is economically significant, although evidence of benefits is lacking (13). We observed a major decreasing trend in the use of cough and cold preparations, which probably indicates better diagnostics and more disease‐specific treatment of cough (Figure 1).

In conclusion, systematic and nationwide public health interventions for asthma, COPD, and allergic conditions have improved diagnostics, early treatment, and awareness of these diseases in Finland. Consequently, the use of medication for respiratory diseases has significantly increased, although the overall burden for patients and society has decreased. Importantly, less symptomatic medication has been consumed for cough and cold. Although improved inhalation therapy has helped patients, such a therapy should be monitored to prevent overuse and overtreatment.

## AUTHOR CONTRIBUTIONS

Vesa Jormanainen, Fredrik Herse, Riikka‐Leena Leskelä, and Tiina Mattila collected and analysed most of the data. Tiina Mattila and Tari Haahtela outlined the first version of the manuscript. All authors interpreted the data, contributed to the writing process, and have read and agreed on the manuscript.

## CONFLICT OF INTEREST STATEMENT

The authors do not have any relevant conflicts of interest concerning the submitted work.

## FUNDING INFORMATION

The Finnish Institute for Health and Welfare, the Hospital District of Helsinki and Uusimaa (HUH), and the Research Foundation of the Pulmonary Diseases funded the collection and analysis of the data and writing the paper.

## Data Availability

Data analysed in this study is not directly available to others. Anybody may apply for a study permit and access to the official data from reported registers. Enquiries should be addressed to the corresponding author, Dr. Tiina Mattila (tiina.m.mattila@hus.fi).
